# Disparities in Advanced Peripheral Arterial Disease Presentation by Socioeconomic Status

**DOI:** 10.1007/s00268-022-06513-0

**Published:** 2022-03-18

**Authors:** Elzerie de Jager, Ronny Gunnarsson, Yik-Hong Ho

**Affiliations:** 1 College of Medicine and Dentistry, The James Cook University, 1 James Cook Drive, Townsville, QLD 4811 Australia; 2 General Practice/Family Medicine, School of Public Health and Community Medicine, Institute of Medicine, The Sahlgrenska Academy, University of Gothenburg, PO BOX 453, 405 30 Goteborg, Sweden; 3 Research, Education, Development & Innovation, Primary Health Care, Region Västra Götaland, Sweden; 4 Primary Health Care Clinic for Homeless People, Närhälsan, Region Västra Götaland, Sweden; 5 Townsville Clinical School, The Townsville Hospital, 100 Angus Smith Drive, Townsville, QLD 4818 Australia

## Abstract

**Background:**

Diabetes and peripheral arterial disease (PAD) often synergistically lead to foot ulceration, infection, and gangrene, which may require lower limb amputation. Worldwide there are disparities in the rates of advanced presentation of PAD for vulnerable populations. This study examined rates of advanced presentations of PAD for unemployed patients, those residing in low Index of Economic Resources (IER) areas, and those in rural areas of Australia.

**Methods:**

A retrospective study was conducted at a regional tertiary care centre (2008–2018). To capture advanced presentations of PAD, the proportion of operative patients presenting with complications (gangrene/ulcers), the proportion of surgeries that are amputations, and the rate of emergency to elective surgeries were examined. Multivariable logistic regression adjusting for year, age, sex, Charlson Comorbidity Index, and sociodemographic variables was performed.

**Results:**

In the period examined, 1115 patients underwent a surgical procedure for PAD. Forty-nine per cent of patients had diabetes. Following multivariable testing, the rates of those requiring amputations were higher for unemployed (OR 1.99(1.05–3.79), *p* = 0.036) and rural patients (OR 1.83(1.21–2.76), *p* = 0.004). The rate of presentation with complications was higher for unemployed (OR 7.2(2.13–24.3), *p* = 0.001), disadvantaged IER (OR 1.91(1.2–3.04), *p* = 0.007), and rural patients (OR 1.73(1.13–2.65), *p* = 0.012). The rate of emergency to elective surgery was higher for unemployed (OR 2.32(1.18–4.54), *p* = 0.015) and rural patients (OR 1.92(1.29–2.86), *p* = 0.001).

**Conclusions:**

This study found disparities in metrics capturing delayed presentations of PAD: higher rates of presentations with complications, higher amputation rates, and increased rates of emergency to elective surgery, for patients of low socioeconomic status and those residing in rural areas. This suggests barriers to appropriate, effective, and timely care exists for these patients.

## Introduction

Diabetes-related peripheral neuropathy and peripheral arterial disease (PAD)-related arterial insufficiency often synergistically lead to foot ulceration, infection, and gangrene, which may require lower limb amputation [1, 2]. Worldwide, there is one diabetes-related lower extremity amputation occurring every 30 seconds [3]. Advanced presentations of PAD indicate decreased access to surgical care.

Internationally, there are disparities in access to PAD surgical services for vulnerable populations. In the USA, studies have demonstrated disparities in the amputation rates among patients with diabetes or PAD by race/ethnicity [4–9], income levels [4–8, 10, 11], insurance status [5, 7], and rurality [8, 9, 12]. Studies conducted in countries with a universal healthcare system have shown similar findings. For patients from low socioeconomic areas, studies have found higher rates of diabetic foot ulceration in Scotland [13] and higher rates of PAD-related amputations for patients in Canada [14], Finland [15], New Zealand [16], Scotland [13], and the UK [17]. In Canada, those residing in rural areas have higher rates of PAD-related amputations [18]. There is a lack of Australian data on access to PAD surgical services for vulnerable populations.

Most studies examining socioeconomic status use neighbourhood-level indices rather than individual-level data. Some Australian hospital databases include a patient reported free text variable on occupation—an important contributing factor to socioeconomic status [19]. This allows examination of access to PAD surgical services by both self-reported individual- and neighbourhood-level indices of socioeconomic status.

There are several ways to potentially capture advanced presentations of PAD utilising administrative data including outcomes like the proportion of patients presenting with complications (gangrene/ulcers), the proportion of surgeries that are amputations, and the rate of emergency to elective surgeries. This study examined how self-reported unemployment, neighbourhood-level socioeconomic status, and rurality are associated to these outcomes in Australia.

## Methods

### Study design

A retrospective study was conducted at a regional tertiary care centre in Queensland, Australia. Due to data availability, a ten-year period between January 2008 and August 2018 was examined. Data for all surgical procedures for PAD in adult (aged > 18 years) patients were extracted. Ethics approval was received from the Townsville Hospital and Health Service Human Research Ethics Committee in Australia (HREC/QTHS/57820). The ethics approval included a patient consent waiver, which was required due to the retrospective design of the study.

### Data sources

Two administrative databases were utilised. The Operating Room Management Information System (ORMIS) was used to extract operative details for surgical procedures performed. ORMIS patient identification numbers were then matched to the Hospital Based Corporate Information System (HBCIS).

### Outcome variables

PAD was selected by principal diagnosis description codes indicating diabetes-related peripheral neuropathy (E10.52, E11.52, E11.51, E11.73, E11.71, E11.69, E11.61, E10.73, E13.73) and PAD (I70.21, I70.22, I70.24, I70.23, I70.20, R02)). Complications were coded by principal diagnosis description codes indicating ulcers or gangrene (I70.24, I70.23, R02, E10.52, E11.52, E11.73, E10.73, E13.73). Amputations were identified by the principal procedure description and included both amputations below (minor) and above (major) the ankle (44,367–00, 44,367–02, 46,480–00, 46,465–00, 44,338–00, 44,358–00, 44,364–01, 44,364–00, 44,325–01).

### Predictors

To code an unemployment variable, a free text occupation variable was used. Responses indicating unemployment were identified, including synonyms/variable spellings of unemployed, and the various names of unemployment social security benefits (Job Seeker, Working for the Dole, and Newstart). Text in the occupation field indicating that patients were not in the labour force (disability support/pensions, stay at home parents, students, retirees, and pensioners) was not coded as unemployed.

Neighbourhood-level socioeconomic status was attained by linking patients’ residential postcodes to 2016 Census tract Index of Economic Resources (IER) data. This proxy for socioeconomic status focuses on financial aspects of socioeconomic advantage and disadvantage [20]. For this analysis, the bottom three IER national deciles, that is, those with the greatest relative lack of access to economic resources, were compared to the top three national deciles.

Residential postcodes were also linked to the Australian Statistical Geography Standard Remoteness Area (ASGS-RA) index [21]. This index divides Australia into five levels of remoteness, based on relative access to services: major cities, inner regional, outer regional, remote, and very remote. For this analysis the five levels were rolled into three: major cities/inner regional, outer regional, and remote/very remote.

The Charlson Comorbidity Index (CCI) was used to adjust for pre-existing comorbidities [22]. Comorbidities included in the index were derived from diagnosis codes assigned during the episode of care using ICD 10-AM (Australian modification) codes [22]. The index includes myocardial infarction, congestive heart failure, PAD, cerebrovascular disease, dementia, chronic pulmonary disease, rheumatic disease, peptic ulcer disease, mild liver disease, diabetes with/without chronic complications, hemiplegia or paraplegia, renal disease, any malignancy including lymphoma and leukaemia (except malignant neoplasm of the skin), moderate or severe liver disease, metastatic solid tumour, and AIDS/HIV. A weighted score was assigned to each comorbidity; a score of zero indicates that no comorbidities were found, and the higher the score, the more the comorbidities were identified.

### Statistical analysis

Independent factors examined were unemployment, low socioeconomic status (IER), and rurality (ASGS-RA). Descriptive statistics was presented as well as simple group comparisons. Pearson’s Chi-squared tests were used for categorical variables while continuous variables were analysed with ANOVA tests.

Multivariable logistic regression models, based on a conceptual model, were used adjusting for age, sex, year of procedure, and CCI as confounding variables. The primary outcomes (dependent variables) were the rate of amputations, advanced presentations (ulcers/gangrene), and the emergency-to-elective surgery ratio.

Sociodemographic variables were included in a subsequent analysis where appropriate. For analysis including the domains of unemployment and IER, rurality was also included in the sociodemographic adjustment models. For analysis of rurality, both unemployment and IER were also included in the sociodemographic adjustment models.

All analyses were performed using Stata 14/MP statistical software package (StataCorp, College Station, TX). All tests were done two-sided. The level of significance was set to 0.05.

## Results

A total of 1116 patients underwent a surgical procedure for PAD between 2008 and 2018 (Table 1). Many patients had diabetes (*n* = 542 (48.6%)). The proportion of surgeries that were amputations was 41.1% (459); 75.2% (345) were minor and 24.8% (114) were major.

**Table 1:** Demographics of study population

Characteristic	Overall *n* = 1,116	Occupation			Index of economic resources			Rurality		
		Other *n* = 1,068 (95.7%)	Unemployed *n* = 48 (4.3%)	*P* value	Advantaged *n* = 268 (24%)	Disadvantaged n = 283 (25.4%)	*P* value	Urban *n* = 145 (13%)	Rural *n* = 88 (7.98%)	*P* value
*Age*										
Mean (SD)	63.1 (13.1)	63.8 (12.7)	45.9 (10.3)	< 0.001	65.5 (12.3)	61.1 (14.76)	0.002	65.4 (11.2)	61.7 (13.6)	0.0573
*Sex, n (%)*										
Male	773 (69.3)	730 (68.4)	43 (89.6)	0.002	193 (72)	174 (61.5)	0.009	121 (83.5)	63 (71.6)	< 0.001
*Charlson Comorbidity Index*										
Mean (SD)	1.72 (1.86)	1.74 (1.87)	1.21 (1.39)	0.0534	1.57 (1.79)	1.89 (1.96)	0.0466	1.23 (1.53)	1.85 (1.8)	0.0035
*Markers of advanced disease at presentation, n (%)*										
Amputations	459 (41.1)	433 (40.5)	26 (54.2)	0.061	83 (31)	133 (47)	< 0.001	29 (20)	44 (50)	< 0.001
Complications	169 (15.1)	163 (15.3)	6 (12.5)	0.602	42 (15.7)	35 (12.4)	0.264	22 (15.2)	9 (10.2)	0.403
Emergency surgery	554 (49.6)	521 (48.8)	33 (68.8)	0.007	127 (47.4)	150 (53)	0.188	54 (37.2)	45 (51.1)	0.006

Unemployed patients comprised 4.3% (48) of cases. Those who were unemployed tended to be younger were more likely to be male, and more likely to require emergency surgery. There was no significant variation in CCI, rates of amputations, or rates of those presenting with complications.

There were 25.4% (283) of patients in the most disadvantaged IER. These patients tended to be younger, were less likely to be male, had a higher CCI, were more likely to require an amputation, and were more likely to be admitted through the emergency room. There was no significant variation in those presenting with complications, or those requiring emergency surgery.

There were 7.98% (88) of patients in the remote and very remote category. These patients were less likely to be male, had a higher mean CCI, were more likely to require an amputation, were more likely to require an emergency surgery, and were more likely to be admitted through the emergency room. There was no significant variation in age or in the rate of those presenting with complications.

### Factors associated with the rate of amputations

There were disparities in the rate of amputations due to PAD for unemployed patients (OR 2.27 (1.21–4.52), 0.011), and for those residing in disadvantaged IER areas (OR 1.93 (1.33–2.81), 0.001) and in rural areas (OR 1.95 (1.46–2.6), < 0.001) (Table 2). The disparity remained statistically significant for unemployed (OR 1.99 (1.05–3.79), 0.036) and rural (OR 1.83 (1.21–2.76), 0.004) domains after adjusting for sociodemographic variables.

**Table 2:** Association between unemployment, socioeconomic disadvantage, rurality, and advanced presentations of peripheral arterial disease

	Unadjusted OR (95% CI), *p* value	Adjusted* OR (95% CI), *p* value	Adjusted also including sociodemographic adjustment** OR (95% CI), *p* value
*Rate of amputations*			
Unemployment	1.73 (0.97–3.1), 0.063	**2.27 (1.21–4.25), 0.011**	**1.99 (1.05–3.79), 0.036**
Low Index of Economic resources (IER)	**1.97 (1.39–2.8), < 0.001**	**1.93 (1.33–2.81), 0.001**	1.47 (0.902–1.17), 0.066
Rural	**2.06 (1.56–2.73), < 0.001**	**1.95 (1.46–2.6), < 0.001**	**1.83 (1.21–2.76), 0.004**
*Rate of presentation with gangrene or ulcers*			
Unemployment	**6.39 (1.97–20.7), 0.002**	**7.67 (2.28–25.7), 0.001**	**7.2 (2.13–24.3), 0.001**
Low IER	**2.74 (1.88–4), < 0.001**	**2.54 (1.68–3.84), < 0.001**	**1.91 (1.2–3.04), 0.007**
Rural	**2.30 (1.71–3.09), < 0.001**	**1.97 (1.45–2.67), < 0.001**	**1.73 (1.13–2.65), 0.012**
*Rate of emergency to elective surgery*			
Unemployment	**2.31 (1.24–4.3), 0.008**	**2.41 (1.23–4.7), 0.01**	**2.32 (1.18–4.54), 0.015**
Low IER	1.25 (0.896–1.75), 0.188	1.25 (0.866–1.8), 0.235	0.909 (0.603–1.37), 0.65
Rural	**1.4 (1.08–1.82), 0.011**	1.26 (0.963–1.66), 0.092	**1.92 (1.29–2.86), 0.001**

*All adjusted analysis adjusted for age, sex, year of procedure and Charlson Comorbidity Index

**For analysis including the domains of unemployment and IER, rurality was also included in the sociodemographic adjustment models. For analysis of rurality, both unemployment and IER were also included in the sociodemographic adjustment models

### Factors associated with the rate of presentations with complications (gangrene or ulcers)

There was a disparity in the rates of presentations with gangrene or ulcers due to atherosclerosis for all domains examined: unemployment (OR 7.67 (2.28–25.7), 0.001), low IER (OR 2.54 (1.68–3.84), < 0.001), and rurality (OR 1.97 (1.45–2.67), < 0.001). After adjusting for sociodemographic variables, the disparity remained statistically significant for all domains: unemployment (OR 7.20 (2.13–24.34), 0.001), low IER (1.91 (1.20–3.04), 0.007), and rurality (OR 1.73 (1.13–2.65), 0.012).

### Factors associated with the rate of emergency to elective surgery

Unemployed patients had higher rates of emergency to elective surgery (OR 2.41 (1.23–4.7), 0.01). This remained significant after adjustment for rurality (OR 2.32 (1.18–4.54), 0.015). Those residing in rural areas had a significant disparity in the rate of emergency to elective surgery after adjustment for unemployment and low IER (OR 1.92 (1.29–2.86), 0.001). There were no significant disparities in emergency to elective surgery rates for those residing in the most disadvantaged IER areas.

## Discussion

This study found disparities in metrics capturing delayed presentations of PAD: higher rates of presentations with complications, higher amputation rates, and increased rates of emergency to elective surgery, for patients of low socioeconomic status and those residing in rural areas. This suggests barriers to appropriate, effective, and timely care exists for these patients.

The outcomes examined in these metrics of advanced presentation for PAD have a significant impact on patient quality of life. In Australia, emergency admissions with PAD have worse post-operative outcomes [23]. Most amputations for PAD are preceded by foot ulceration [24], and lower limb amputations are associated with significantly decreased quality of life, increased morbidity, mortality, and healthcare costs [25].

There were disparities in all markers of PAD access to surgical care for patients residing in remote or very remote areas. In Australia, just as globally, those residing in rural areas tend to have higher adverse health outcomes, including both higher hospitalisation and mortality rates for chronic diseases, and poorer access to and use of health services [26, 27]. There are several factors that may contribute to this disparity: lower socioeconomic status, a higher prevalence of lifestyle risk factors, lower access to primary care services, and a relative shortage of healthcare professionals—particularly specialists [24, 26, 27]. However, geographical proximity to primary care or hospital clinics was not associated with diabetic foot ulcers or lower extremity amputation in a UK cohort study [28]. Low health literacy may also contribute; one Australian study of diabetic patients attending rural podiatry clinics found a lack of knowledge of basic diabetic information, indicating insufficient provision of effective educational services for patients in regional and rural areas [29].

There were disparities for patients of low socioeconomic status, captured by both self-reported unemployment as well as the neighbourhood-level IER. These results mirror those of another Australian study, conducted in the state of Victoria, which found that patients with diabetes residing in low socioeconomic status areas were more likely to have foot ulceration and below knee amputation [30]. Among the most deprived within a population, the incidence of disease tends to be higher, and its subsequent outcomes tend to be worse—a phenomenon known as the social gradient of health [31]. People who are socioeconomically disadvantaged in Australia have higher rates of several risk factors for PAD including higher rates of cardiovascular disease, diabetes, and chronic kidney disease [32]. This study adjusted for the CCI; comorbidities in this index include a history of myocardial infarction, congestive heart failure, diabetes, and renal disease. Despite adjusting for the CCI, low socioeconomic status was associated with advanced presentations of PAD.

A high percentage of patients with PAD-related surgery had diabetes. Studies have shown that a decrease in PAD amputation rates is possible, regardless of the presence of comorbidities, with appropriate primary care (aggressive medical care of hypertension, hyperlipidaemia, and diabetes), risk factor modification, timely referral to vascular surgical centres, adequate foot and wound care, and aggressive revascularisation therapies [33–37]. Primary care is vital compliance to pharmacotherapy, including antiplatelet and statin therapy, lowers the risk of amputation and the mortality for patients with PAD [38–40]. Similarly, smoking cessation increases the amputation-free survival time and decreases mortality rates [41].

### Upstream disparities

While these metrics specifically examine surgical care, disparities in the metrics do not solely represent a lack of access to surgical care, rather a lack of access to health care more broadly. In the USA, one study in a hospital outpatient setting for PAD found that only 33% of patients received antiplatelets, 33% received statins, and 36% of smokers received smoking cessation counselling [42]. In another study, 32% of patients who underwent an extremity amputation for PAD did not receive any arterial testing (i.e. ankle–brachial index, imaging, or angiography) in the year before amputation—there was therefore no investigation into the possibility of revascularisation, which may have prevented the need for an amputation [43]. These are just two examples of studies demonstrating missed opportunities to alter the path of PAD progression. There are likely multiple upstream disparities in health care that ultimately result in a disparity in these surgical metrics.

### Limitations

Unemployment was used as a marker for low socioeconomic status at an individual rather than neighbourhood characteristic level. In Australia, aged pension benefits are available from 66 years of age. The mean age of admission for surgical patients with PAD in this cohort was 69.3 years—once many people have left the labour force. Despite this, unemployment was a sensitive marker for disparities in advanced PAD presentation, both prior to and after adjustment for a patient’s age.

To attain data on neighbourhood-level socioeconomic status, residential postcodes were linked to census tract IER data. More granular geographical areas called statistical local areas (SLA) can be linked to census tract data. These granular areas perform more consistently as area-based markers of socioeconomic status when compared to postcodes [44, 45]. The IER may have been a more reliable indicator of socioeconomic status had the data been linked using a patient’s SLA.

Aboriginal and/or Torres Strait Islander Australians are more likely to require lower limb amputations for indications of PAD, diabetic foot ulcers, or sepsis [46–50]. As such, adjusting for Aboriginal and/or Torres Strait Islander status when examining unemployment, IER and rurality, as well as examining outcomes for this group individually, would have been prudent in this study. Unfortunately, the Queensland Government did not allow for the acquisition of any data relating to Aboriginal and/or Torres Strait Islander status, despite investigators acquiring the necessary full Human Research Ethics Committee approval and attaining specific extramural funding to analyse this data.

## Conclusion

This study found disparities in metrics capturing delayed presentations of PAD: higher rates of presentations with complications, higher amputation rates, and increased rates of emergency to elective surgery, for patients of low socioeconomic status and those residing in rural areas. This suggests barriers to appropriate, effective, and timely care exists for these patients.
